# Aging Effects of Aqueous Environment on Mechanical Properties of Calcium Carbonate-Modified Epoxy Resin

**DOI:** 10.3390/polym12112541

**Published:** 2020-10-30

**Authors:** Anna Rudawska, Mariaenrica Frigione

**Affiliations:** 1 Faculty of Mechanical Engineering, Lublin University of Technology, Nadbystrzycka 36 Str, 20-618 Lublin, Poland; a.rudawska@pollub.pl; 2 Department of Innovation Engineering, University of Salento, Via Arnesano, 73100 Lecce, Italy

**Keywords:** epoxy resin, calcium carbonate, aqueous environment, mechanical properties

## Abstract

The purpose of this study was to assess the effects of different aqueous environments (i.e., demineralised, distilled and spring water) on the mechanical properties of a cold-cured bisphenolic epoxy resin modified with the addition of calcium carbonate filler, typically employed as structural adhesive. The parameters selected for the analysis have been; the kind of curing agent employed to cure the epoxy resin at ambient temperature (i.e., Mannich base and triethylenetetramine); the load of calcium carbonate added to liquid epoxy (i.e., from 1 to 3 g per 100 g of resin) and; the duration of the exposure to the different aging conditions (i.e., from 1 to 10 months). Cylindrical specimens of calcium carbonate-modified epoxy systems were tested in compression mode, before and after each of the aging regimes. The effect of the selected curing agents is very small, and they are both suitable for a cure at ambient temperature, on the unfilled epoxy on compressive maximum strength and strain at break; the choice of the hardener affects instead the compressive modulus. The CaCO3 amount was demonstrated to have a significant effect on the mechanical characteristics of un-aged epoxy systems, leading to growth in compressive modulus and maximum strength with reductions in strain at break. Generally speaking, the aging time noticeably affects the compressive properties of calcium carbonate-modified epoxies while almost negligible is the kind of water employed in each exposure regime. Notwithstanding the adverse effects of an aqueous environment on compressive mechanical properties of CaCO_3_-filled epoxies, these systems keep compressive modulus and maximum strength greater than, and close to, respectively, the same characteristics measured on unaged unfilled control epoxies, demonstrating the positive effect of the addition of this kind of filler to epoxy-based structural adhesives, especially with the addition of 2 and 3 g of CaCO_3_ per 100 g resin. The results obtained in this study demonstrated that it is possible to contrast the detrimental effects observed in cold-cured epoxy-based structural adhesives due to their aging in water upon the addition of limited amounts (particularly at 2 and 3 g per 100 g resin) of a cheap CaCO_3_ filler.

## 1. Introduction

Epoxy resins are compounds with a broad applicability, which stems from their outstanding properties, in particular, their high mechanical strength, good chemical resistance and thermal stability, and excellent adhesion to different substrates [1,2,3]. Epoxy resins, in fact, are widely used as adhesives and sealing materials; they can also effectively serve as matrices for fibre reinforced polymer (FRP) composites [4].

The main advantages of epoxies over other thermosetting resins reside in their low shrinkage displayed during curing, in the possibility to process (i.e., “cure”) the resins in different environmental conditions (namely, heat- or cold-cure) and to properly modify their (chemical, mechanical, functional) properties within a broad range of values. All these features are achieved through an accurate selection of the type and amount of the curing agent, type and quantity of modifiers, as well as curing and aging conditions [3,5,6,7,8,9,10].

For the most common applications, the durability of the epoxy in the specific service conditions plays a crucial role for the success of its usage. Epoxy compounds are usually characterised by resistance to water and to many solvents (i.e., petroleum, oil, organic solvents, diluted acids and bases) (references). On the other hand, when employed in particular conditions, they can be severely affected by stronger chemicals (such as, acetone, benzene, trichloroethylene, dimethylformamide, concentrated acids, and ammonia) and by temperatures exceeding 50 °C [11,12,13]. The properties and performance of an epoxy adhesive/matrix resin, strongly depend on the curing conditions, i.e., the conditions employed to carry on the cross-linking reactions of the resin, and on the service conditions, comprising the environment of exposure during the whole working-life. [1,9,14,15,16,17].

In order to modify the properties and characteristics of an epoxy, and produce compounds suitable for specific needs, different routes can be selected. A *chemical modification* of the epoxy resin and/or of its curing agent can be accomplished for a particular purpose: To achieve, for instance, an enhancement of thermal resistance, an increase in toughness, or a reduction in curing time [2,11]. Physical modification is a simple and cheap method involving the addition, during processing, of different (organic, metallic, ceramic, etc.) compounds in the liquid resin able to change one or more features of the epoxy [1,11]. Finally, *physico-chemical modification* combines the two routes previously described. By using this method, the properties of an epoxy compound can be adjusted with an even greater precision [18,19].

Although, inorganic fillers are mainly introduced to reduce the formulation costs, they can be added to adjust the viscosity of the resin, to tune its density, to increase their toughness, acting as reinforcing agents restricting the flexibility and the mobility of epoxy chains. Based on their chemical and physical features, the presence of specific fillers in epoxies can further reduce the shrinkage during curing, improve their thermal/fire resistance and the resistance to specific environments, possibly reduce sensitivity to moisture, introduce some electric conductivity, or merely change the colour/transparency of the epoxy [20,21,22]. The size and shape of such particles, as well as their degree of dispersion in the epoxy, dramatically affect the final properties of the cured composite system [23,24].

The use of calcium carbonate in epoxy resins has been the subject of several studies [25,26,27,28,29,30]. Among the others, CaCO_3_ filler is the more employed in epoxy (adhesives, coatings and paints, components for various applications, matrices for FRP) due to its very low price, a wide availability, its thermal stability and insolubility in water, biocompatibility characteristics. Independent studies focusing on epoxy-based fibre reinforced composites found that the addition of CaCO_3_, in the form of micrometric particles, leads to enhanced superficial properties (i.e., scratch-resistance and hardness), mechanical strength and flexural modulus, corrosion resistance and thermal stability [31,32,33]. Composites based on epoxy resin containing CaCO_3_ (average size around 57 μm, filler content in the range 5–15 wt.%) have been proposed to realize biocompatible implants, for instance in the bone reconstruction [34]. Medium contents of CaCO_3_ filler (i.e., 15 wt.%) in epoxy were able to increase tensile modulus, decrease both the tensile and flexural strengths of the neat resin, as well as the strain at break [35]. Chen and co-workers [28] added an extensive amount (i.e., 60 wt.%) of calcium carbonate to produce epoxy-based coatings. It has been also found that the addition of CaCO_3_ (30 to 60 in mass parts) to epoxy films leads to higher values of Young’s modulus and Shore D hardness with respect to un-filled epoxy [29]. Medium content (i.e., 10–20 wt.%) of calcium carbonate can be effective to enhance the corrosion resistance of an epoxy paint for carbon steel [36]. This content can be further lowered (down to 3 wt.%) if corrosion inhibitors are employed to modify the surface of calcium carbonate particles [37]. Most of the reviewed studies have reported results collected for heat-cured epoxies (i.e., curing temperatures range from 120 °C to 150 °C, often completed by an additional post-cure step around 220–240 °C).

As previously outlined, the epoxies are severely affected by the environmental conditions that characterize their curing process as well as their whole service life. In particular, if it is expected that an epoxy will be subjected to an aggressive environment during its service life, or it is merely exposed outdoors, it is necessary to analyse the effect of the specific service environment on its characteristics, even at different exposure times [14,16,17,20,38,39]. The presence of humidity or liquid water is considered one of the most detrimental environment that can be encountered by structural adhesives. Due to the presence of the cross-linked structure of polar groups, which are able to attract and bond water molecules, epoxy resins are prone to absorb great amounts of water. Once water (present in common outdoor environments as moisture, rain, water of lakes or rivers, etc.) enters epoxies, it can lead to undesirable reductions in glass transition temperature (Tg), as well as in stiffness and strength and bond characteristics, due to the so-called plasticization phenomenon; as a consequence, the epoxy adhesive can experience a marked decrease of its load-bearing capacity [9]. 

The effects of immersion in water on mechanical properties of filled epoxies are affected by the size and the kind of the filler, and by its content in the resins; the behavior of water absorption in epoxy can be also influenced by the filler-matrix interactions. In this regard, Sugiman, Salman and co-workers published several papers on the effect of calcium carbonate particles on mechanical characteristics of epoxy resins aged to water [40,41,42]. They found that the addition of medium amounts (7–46 wt.%) of this filler (with an average size of 15 μm) decreased the water uptake at saturation of the modified epoxy in comparison to neat resin, due to the more tortuous path for water ingress in presence of particles, the higher the CaCO_3_ content, the lower the value of water at saturation. Accordingly, the expected reductions in tensile strength and modulus, due to the wall known plasticization effects, was lower than tensile strength observed in un-filled epoxy. Referring to the properties in dry conditions, the tensile strength of CaCO_3_-filled epoxy was lower and the elastic modulus higher than unfilled epoxy; the fracture toughness of filled epoxies increased compared with the neat epoxy. The same property increased upon immersion in water for both pristine and CaCO_3_-filled epoxy. 

Since no systematic studies aimed at assessing the effects of the presence of CaCO_3_ particles on the mechanical properties, calculated in compression mode, of epoxy adhesives aged in aqueous environments are currently available in literature, the present research want give a contribution on this topic. To this end, a cold-cured structural adhesive based on epoxy resin, modified with the addition of small amounts of calcium carbonate, was subjected to three different aging procedures in aqueous environments. The effect of such aging environments on mechanical (compressive) properties of the filled epoxy was assessed at different immersion times. The durability characteristics of epoxy adhesives offering high compressive strength is, in fact, a critical issue for the success of high performance structural bonding applications, such as in automotive, aerospace, electronics, and electrical industries.

## 2. Experimental 

### 2.1. Materials and Production Of Epoxy Formulations

Different formulations based on a diglycidyl ether of bisphenol A (DGEBA) epoxy resin (commercialised by CIECH Resins, Nowa Sarzyna, Poland, with the trade name Epidian 5), were analysed in the present study. This epoxy is manly employed as cold-curing adhesive to join diverse materials (i.e., glass, metals, ceramics, thermosetting polymers), as a primer for concrete substrates, as sealant and coating, as matrix to produce FRP composites. The resin, whose main properties are summarized in Table 1, has an epoxide equivalent weight of 196–208 g/mol. 

The pristine epoxy resin was cured using two curing agents. The first one was a Mannich base curing agent (supplied by CIECH Resins, Nowa Sarzyna, Poland, with the trade name TFF). Mannich base is a cheap curing agent for epoxies, indicated for the low temperature cure processes, such as in the case of adhesives. TFF has an amine number of 500–700 mg KOH/g; some of its properties are summarised in Table 2.

An unmodified Epidian 5-based formulation was produced with TFF curing agent, representing the reference for other modified systems realized with the same hardener. The composition of this formulation was set at 100:26 resin:curing agent (in weight), corresponding to the stoichiometric epoxy/amine molar ratio. This control formulation will be indicated as E5/TFF/100:26. A second curing agent was selected, i.e., triethylenetetramine (manufactured by Ciech-Sarzyna, Nowa Sarzyna, Poland, with trade name Z-1). It is another typical curing agent employed for the cold-curing of the epoxy resins in applications where the supply of any source of heat is impracticable (for instance, as adhesives, matrices for FRP or coatings in infrastructure field [27]. Z-1 curing agent, whose main properties are summarized in Table 3, has an amine number of 1100 mg KOH/g.

The Z-1 curing agent was added to Epidian 5 to produce a second control formulation, indicated as E5/Z-1/100:12. The composition of this formulation was set at 100:12 resin:curing agent (in weight), corresponding to the stoichiometric epoxy/amine molar ratio.Both control formulations, i.e., E5/TFF/100:26 and E5/Z-1/100:12, were modified by adding different amounts (up to 3 g per 100 g of epoxy resin) of calcium carbonate (CaCO_3_) powder, commercialized by Carl Roth GmbH + Co KG, Karlsruhe, Germany, with the trade name E170. The average particle size value is 0.87 μm +/−0.1 μm. The compositions of control and modified epoxy compounds are presented in Table 4.

**Figure 1:**
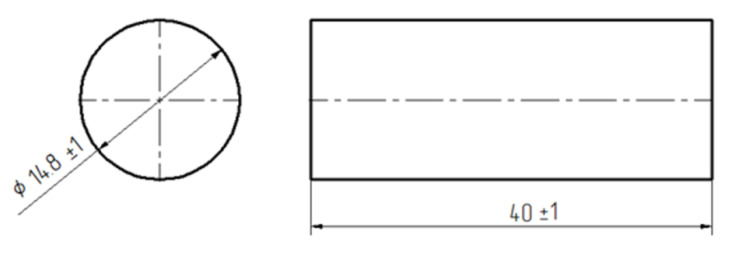
Dimensions [mm] of adhesives sample.

**Figure 2:**
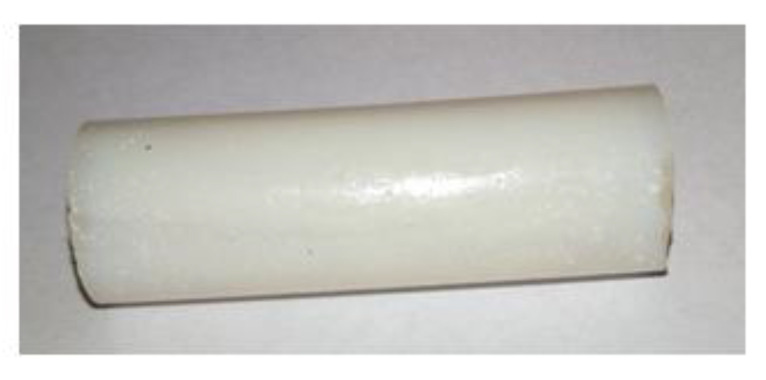
Specimens of E5/Z-1/100:12 epoxy compound after milling procedure.

After weighing the different chemical substances according to the compositions reported in Table 4 (employing a laboratory balance OX-8100, FAWAG S.A., Lublin, Poland), the compounds were mechanically mixed using an anchor stirrer at a speed of 460 RPM. The mixing continued for 2 min; then, the formulations were kept under vacuum for a further 2 min, in order to remove gases and avoid the formation of bubbles. Laboratory conditions were employed for the production of the epoxy formulations, i.e., 22 ± 1 °C and 24 ± 4% R.H. The liquid formulations were, then, poured in polymeric (polypropylene) cylindrical moulds with a volume of 10 mL. The internal surface of the moulds was coated with an anti-adhesive agent (SOUDAL Joint Finish, Soudal NV, Tornhout, Belgium), sprayed for 10 s from a distance of 30 mm. The different formulations placed in the moulds were subjected to a single-stage cure process carried out at an ambient temperature of 22 ± 1 °C, with relative humidity of 24 ± 4%. The curing time was 7 days, as suggested by suppliers and experienced in previous studies [5,8,14]. After this period, the specimens were demoulded and milled, in order to rectify the bases of cylindrical specimens. This process was performed with a FNR26 vertical milling machine no. 629. The employed milling parameters were: spindle speed of 825-1150 RPM and feed 52–74 mm/min. In this way, samples compliant with PN-EN ISO 604-2006 were obtained, whose dimensions and shape are shown in Figure 1 and Figure 2.

For each control or modified compound, 54 specimens were produced, in order to have 6 samples for each composition (reported in Table 4) and for each of the 3 different aging conditions and each of the 3 aging times, plus 6 unaged specimens (as control samples) for each system employed to measure the initial properties. The total number of the produced samples was 480.

### 2.2. Aging Procedures 

Samples of cold-cured epoxy compounds containing different amounts of CaCO_3_ were placed in three different aqueous environments, i.e., demineralised, distilled, and spring water, and kept for different time spans: 1, 6, and 10 months, resulting in a total of 9 aging conditions (as reported in Table 5). Each aging set consisted of 6 samples for each formulation. Due to a wide variety of applications where epoxy resins, employed as structural adhesives, can be exposed to freshwater (moisture, rain, water of wetlands, lakes and ponds, etc.), the selection of the aging media was based on the willingness to analyse the effect of pure water on cold-cured epoxy resins. The research aimed to investigate also if and how slightly different compositions of an aqueous non-saline environment can affect the mechanical characteristics of the resins.

*Demineralised water* was obtained in a reverse osmosis process; it contains no mineral salts. It is a chemically pure water (pH = 7) even though, due to the contact with the carbon dioxide of air, the final pH of the water was 6, as tested with a litmus paper.

*Distilled water* has similar properties to demineralised water, although it is not as “pure”. It is obtained through a distillation process. At a room temperature, this water shows a pH of 6–7.

As *spring water*, the still water produced by the Gardinia Aleksandria Company (Poland) was used. It is a drinkable water with a dissolved mineral component content of 273.56 mg/L; the detailed composition of this water is reported in Table 6. The pH value of the spring water was 7.

### 2.3. Mechanical (Compressive) Tests 

The control and modified epoxy samples, cold-cured for 7 days as previously described, were subjected to mechanical tests in compression mode. As these structural adhesives must withstand exposure to various environmental conditions, the mechanical tests in compression mode were performed also on specimens of CaCO_3_-filled formulations subjected to the described aging procedures.

The tests were performed using a Zwick/Roell Z150 machine (a hydraulic-powered machine with a maximum destructive force of 150 kN), in accordance with PN-EN ISO 604 standard. A special holder was inserted between the plates of the instrument, allowing cylindrical samples to be held firmly in plates and vertically loaded.

The compressive tests were performed at room temperature, at a test speed of 10 mm/min. The initial force was set at 100 N. Compressive strength [MPa] and modulus [MPa], and compressive strain [%] were calculated through the mechanical tests, averaging the results obtained on 6 specimens for each set of formulations/conditions. The experimental data were subjected to a basic statistical analysis, eliminating the outermost results; *Statistica* software was employed.

## 3. Results and Discussion

### 3.1. Un-Aged Epoxy Samples Filled with Different Amounts of CaCO_3_

**Figure 3:**
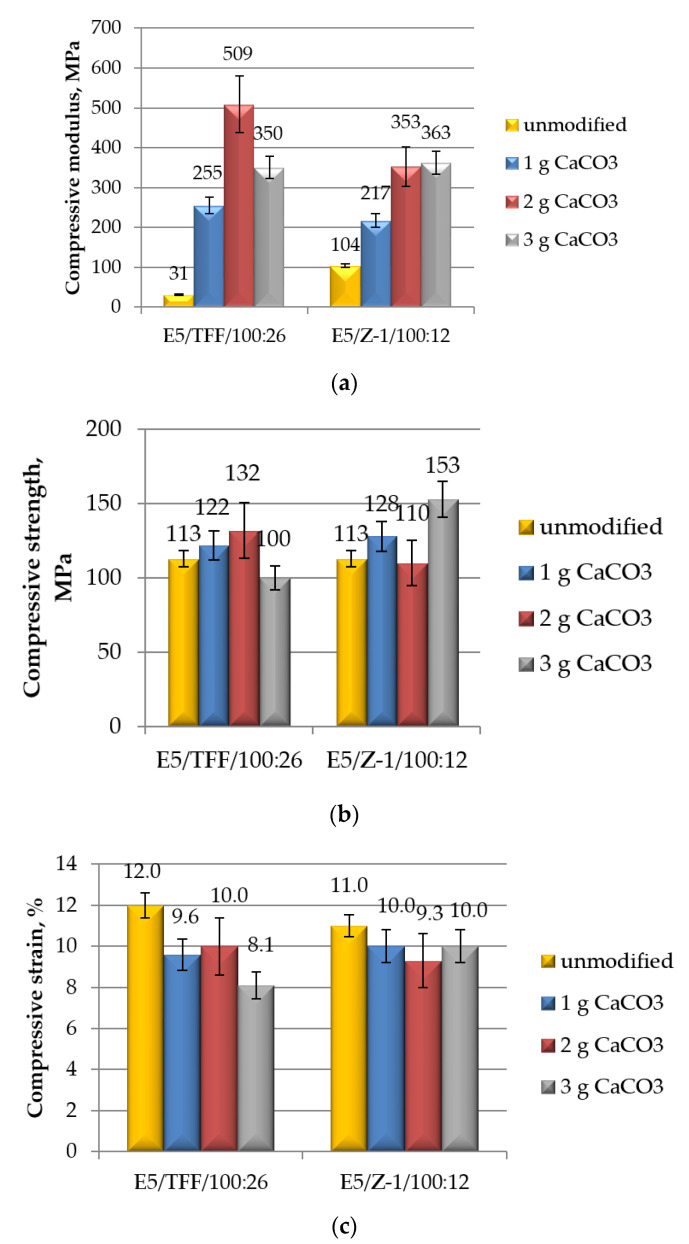
Results of mechanical (in compression mode) tests performed on un-aged control specimens, i.e., E5/TFF/100:26 and E5/Z-1/100:12, and on un-aged epoxy compounds filled with different amounts of CaCO_3_: (**a**) compressive modulus; (**b**) compressive maximum strength; and (**c**) compressive strain at break.

The results, in terms of modulus (Figure 3a), maximum strength (Figure 3b) and strain at break (Figure 3c), of the mechanical (in compression mode) tests performed on un-aged E5/TFF/100:26 and E5/Z-1/100:12 specimens are reported in Figure 3. 

First, we analyse the effect of the curing agent. From the observation of the data reported in Figure 3, it can be concluded that maximum strength and strain at break measured in compression mode are not substantially influenced by the type of hardener. The selection of the curing agent suitable for the cure at ambient temperature, on the other hand, remarkably affects the compressive modulus of the epoxy. The obtained results are in a good agreement with mechanical properties, measured in compression mode, reported for commercial cold-cured epoxy-based structural adhesives. 

The effect of the filler on the mechanical properties of the epoxy resins is analysed next. Generally speaking, the addition of calcium carbonate to the adhesives brings about an increase in both compressive modulus and maximum strength, and a decrease in strain at break, having the CaCO_3_ content a certain influence on the analysed properties.

In particular, in the systems cured with TFF curing agent, i.e., those displaying the lowest value of compressive modulus, the addition of a limited amount (2 g) of the filler leads to a noticeable increase in this characteristic (Figure 3a). However, by increasing further the CaCO_3_ content, the compressive modulus is reduced, even though it results always much higher than that measured on un-filled resin cured with the same curing agent (i.e., E5/TFF/100:26 system).

The growth in modulus of E5/Z-1/100:12 compound is more regular upon the addition of increasing amounts of calcium carbonate, although the increases measured at 2 and 3 g of CaCO_3_ are very close (Figure 3a). However, the standard deviation value measured for this set of specimens (CaCO_3_ content equal to 2g) was slightly higher with respect to the others.

In comparing the two groups of compounds, i.e., cured with TFF, or Z-1 curing agents, respectively, the highest value of compressive modulus (i.e., 509 MPa) was observed for the system E5/TFF/100:26 modified with 2 g calcium carbonate, the lowest (217 MPa) for E5/Z-1/100:12 compound containing the lowest amount (1 g) of this filler. The effect of the filler on this characteristic is similar for both epoxy adhesives at the greatest loads (i.e., 3 g).

The observed appreciable growth in compressive modulus as a result of the addition of small amounts of calcium carbonate reflects what previously found by testing CaCO_3_-epoxy specimens in tensile mode [29,40,41,42]. This result was attributed to the elastic modulus of the filler, which is much higher than that of the epoxy matrix, according to the “rule of mixture” for composite materials [40,42].

In the case of E5/TFF/100:26 systems, the addition of 1 and 2 g of CaCO_3_ leads to a steady increase in maximum strength (Figure 3b); at the highest analysed amount of filler (3 g), on the other hand, a decrease in strength is observed, down to a value even lower than that measured on the un-modified resin. A higher spread of values was observed in the case of the compound including 2 g of calcium carbonate.

The addition of small contents (1 and 2 g) of CaCO_3_ in E5/Z-1/100:12 compounds produces limited variations in maximum strength (Figure 3b), considering the variability of results; an appreciable increase in this characteristic was, conversely, measured at the highest content (3 g) of this filler. This latter is the greatest value of compressive strength (153 MPa) found analysing the filled systems based on the two different curing agents.

It has been reported in previous literature [29,40,41,42] that lower values of tensile and flexural strengths with respect to the pristine resin are obtained upon addition of the same micro-filler to epoxy. This result was mainly attributed to the weak interface strength developed between the matrix and the filler particles [42]. In the reviewed studies, significantly greater amounts of calcium carbonate were added to epoxy adhesives. Apart from this distinct feature, it is likely that the effect of CaCO_3_ filler is unlike when the epoxy is subjected to a compressive load, since the adhesion at the interface filler/matrix probably plays a less primary role.

Finally, the compressive strain of E5/TFF/100:26 systems are reduced upon addition of different amounts of CaCO_3_, being the reductions of this characteristic similar at 1 and 2 g loads. A greater decrease in strain is observed at the highest amount of filler (3 g). The reductions in compressive strain seem to be almost independent by the content of calcium carbonate in the case of E5/Z-1/100:12 compounds. 

Limited reductions in ultimate strain of CaCO_3_-epoxy with respect to the unfilled resin measured in tensile mode were reported also elsewhere [29,40,41,42]. This result, consistent with the increase in elastic modulus, was explained in terms of a stiffening and embrittlement of the epoxy upon addition of the filler [29]; also the irregular shape of the calcium carbonate particles can affect elongation at break to a certain extent [41].

### 3.2. Epoxy Samples Filled with Different Amounts of CaCO_3_ and Aged in Demineralised Water 

**Figure 4:**
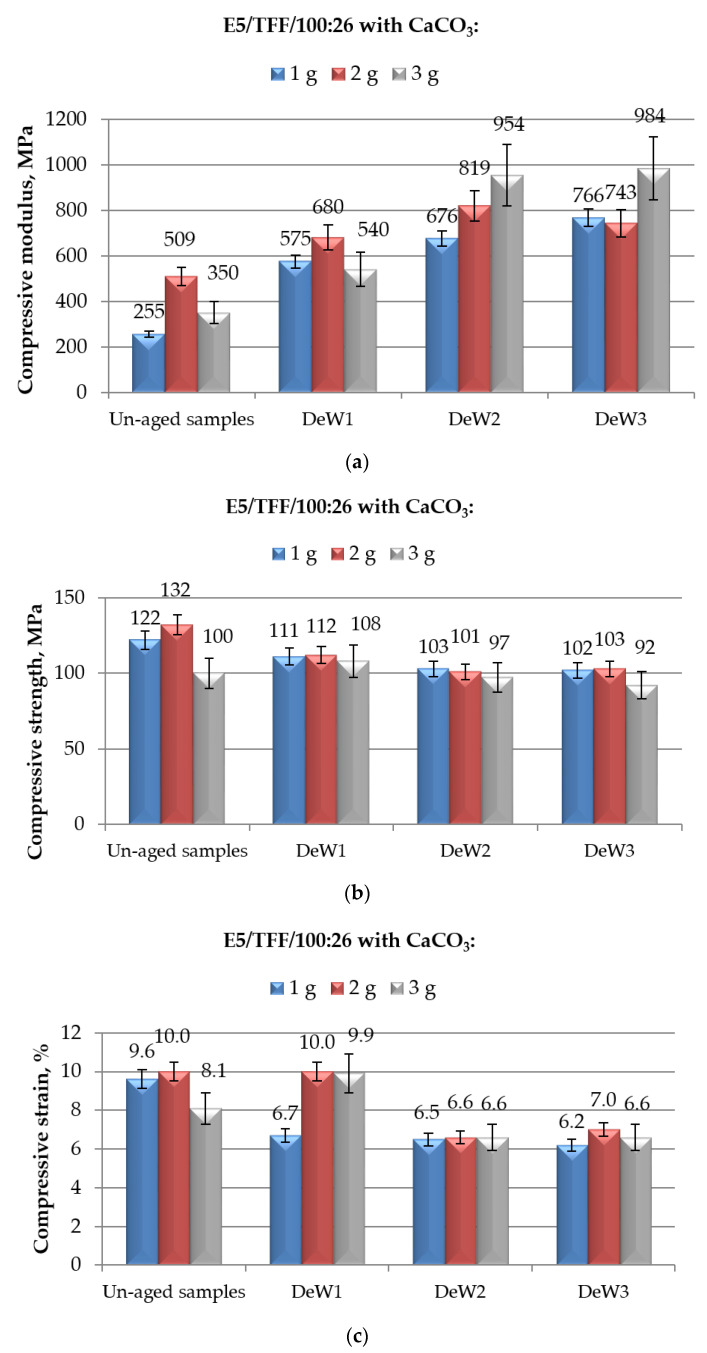
Results of mechanical (in compression mode) tests performed on E5/TFF/100:26 epoxy compounds filled with different amounts of CaCO_3_ and aged for different time spans (from 1 to 10 months) in demineralised water: (**a**) compressive modulus; (**b**) compressive maximum strength; and (**c**) compressive strain at break.

**Figure 5:**
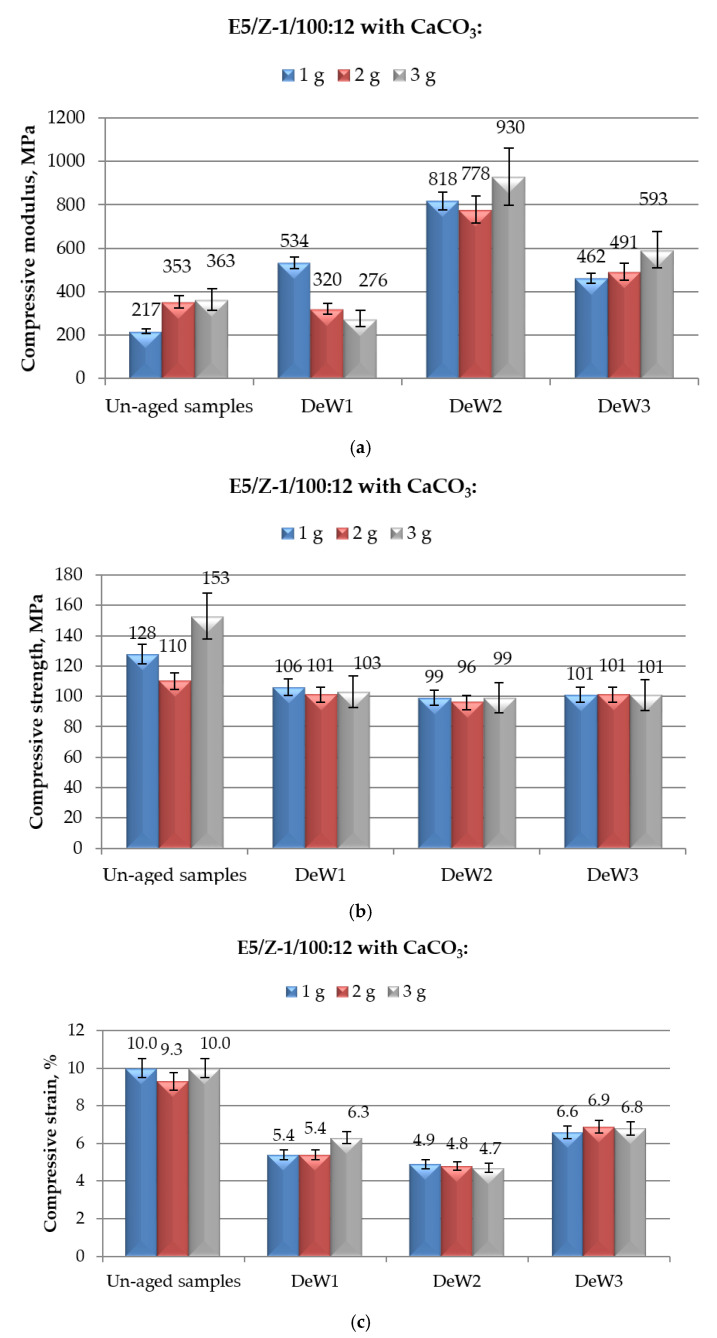
Results of mechanical (in compression mode) tests performed on E5/Z-1/100:12 epoxy compounds filled with different amounts of CaCO_3_ and aged for different time spans (from 1 to 10 months) in demineralised water: (**a**) Compressive modulus; (**b**) compressive maximum strength; and (**c**) compressive strain at break.

The main aim of the present study was the assessment of the effects of an aqueous environment on the mechanical (measured in compression mode) properties of cold-cured epoxy-based structural adhesives in which different amounts of CaCO_3_ filler are added. To this aim, the effect of an immersion in demineralised water was first analysed, and the results are reported in Figure 4 and Figure 5 for the systems based on E5/TFF/100:26, and E5/Z-1/100:12, respectively.

In the case of E5/TFF/100:26 adhesive, filled with different amounts of calcium carbonate, the compressive modulus, that was already greater than that measured on un-filled resin (i.e., 31 MPa), experienced a further increase upon immersion in demineralised water, even if aged for prolonged times, as reported in Figure 4a. The increase was particularly remarkable (+181% over un-aged specimens with the same composition) at the highest amount of calcium carbonate (i.e., 3 g) when the specimens were aged for 10 months, although a significant spread or results was measured for this system. Nevertheless, the trend of mechanical data is clear, and it represents an important advantage for outdoor exposed structural adhesives that can be frequently exposed to moisture/liquid water, even for prolonged periods of time.

As already described, the exposure to freshwater severely affects the characteristics and the behaviour of epoxy resins due to the plasticization of the resin. Plasticization was found to produce severe reductions in elastic modulus measured in tensile mode of both neat and CaCO_3_–filled epoxies [40,41,42]. The detrimental effects of immersion in water on modulus was found to be less severe in the case of calcium carbonate-filled epoxies if compared with neat resin, being attributed to the ability of the micro-particles to hinder the elastic deformation of the matric resin [40,41]. The results reported in previous studies, on the other hand, cannot be directly applied to the mechanical characteristics measured in compression mode, due to the different mechanisms taking place in the resin during the application of tensile versus compressive loads. Additionally, we cannot exclude that a concurrent advancement of curing occurs during the immersion in demineralized water; the specimens, in fact, were cold-cured for a week, which is a time far to be sufficient to achieve the completion of curing carried out at ambient temperature [12,13,43]. A further increase of the degree of cure at longer times is likely to produce a growth in compressive modulus, able to counteract the plasticization effect of water, as previously observed for cold-cured unfilled epoxies tested in tensile mode after different immersion times in demineralised water [44]. These aspects, therefore, deserve further investigation.

Generally speaking, a slight reduction in the compressive strength of CaCO_3_-filled epoxy was observed as a consequence of the exposure to demineralised water, as shown in Figure 4b. The decrease was more evident (up to 23%) for the system E5/TFF/100:26/2CaCO_3_, i.e., the compound showing the highest value of maximum strength among unfilled and modified formulations, while it was contained for the other filled systems (around 8–16%). The immersion time in demineralised water had a certain effect on this characteristic up to 6 months of aging; beyond this time, it becomes almost negligible, taking into account the deviation of results. Comparing the results of filled-systems aged for different times in water with those relative to unfilled unaged E5/TFF/100:26, reported in Figure 3b, it can be concluded that, although aged in demineralised water, the CaCO_3_-filled compounds are able to keep values of compressive strength close to those measured on unaged E5/TFF/100:26 control system, especially at 1 and 2 g loads.

Plasticization related to the ingress of water in unfilled and CaCO_3_-filled epoxies was found to produce substantial reductions also in the tensile strength [40,41,42,43]. It is suggested that, in addition to the plasticization of the matrix, in the presence of a micro-filler water, it can also affect the filler-matrix interface, thus further reducing the resistance to a tensile load [40,41]. The reductions in compressive strength found in the present study, however, are somewhat limited (never exceeding 25%). Once again, a compensatory effect cannot be excluded, given the continuation of curing during the aging in demineralised water, as observed for the tensile strength measured on cold-cured unfilled epoxy immersed for prolonged times in water [44]. 

The behaviour of the strain at break measured in compression mode on epoxy systems loaded with calcium carbonate, reported in Figure 4c, was strongly dependent on the filler content and on the duration of aging. At the lowest content of CaCO_3_, a decrease in strain of about 30% is recorded after only 1 month of immersion; further reductions in strain of this compound due to prolonged aging are limited. On the other hand, the system E5/TFF/100:26/2CaCO_3_ kept the same value of strain at break in compression after the shortest immersion time (1 month); this value was appreciably reduced (up to 34%) at longer immersion times, i.e., after 6 and 10 months. Finally, the compound containing the highest amount (3 g) of filler experienced an initial increase (22%) in strain after a 1-month immersion; then a reduction in this property of about 18% with respect to unaged system was measured. Strong reductions in strain can be calculated for filled systems as a consequence of the immersion in water if compared to unaged E5/TFF/100:26 control system.

In contract, the effect of immersion in water on strain at break on CaCO_3_-filled epoxies was established, and measured in tensile mode, with increases in this characteristic due to the aqueous aging [40,41,42]. This result was attributed to an enhancement in ductile behaviour, due to the plasticization. Possibly, the aging in water caused an appreciable decrease in the glass transition temperature, even around the aging temperature, shifting the epoxy from a glassy state to a rubbery state [42]. It was also hypothesized, however, that the calcium carbonated particles, able to hinder to a certain extent the water ingress, limited the deformations of the filled epoxy [41]. The plasticization, in fact, affected this property to a minor extent than in the case of unfilled resin. In addition, the irregular shape of the CaCO_3_ particles also have an influence, contributing to reduce the deformability of the matrix [41]. As already underlined, the mechanisms taking place in the epoxy during the application of tensile load are likely to be very different from those occurring during a compressive test; this could justify the observed diverse effects on strain at break measured in compression mode of the aging in demineralized water.

The results of mechanical properties measured in compression mode on CaCO_3_-filled E5/Z-1/100:12 compounds aged for different periods in demineralised water are showed in Figure 5. The reported data are roughly in line with those observed on E5/TFF/100:26, with a certain increase in compressive modulus upon immersion, limited reductions in maximum strength and remarkable drops in strain at break. The same observations reported for the CaCO_3_-filled E5/TFF/100:26 can be here repeated to comment the observed results. 

Analysing in detail the data relative to compressive modulus, reported in Figure 5a, the greatest increase in compressive modulus with respect to unaged specimens was observed when the systems were immersed in demineralised water for 6 months, i.e.,: +277%, +120% and +156% for E5/Z-1/100:12/1CaCO_3_, E5/Z-1/100:12/2CaCO_3_ and E5/Z-1/100:12/3CaCO_3_, respectively. At the longest immersion time tested, all the CaCO_3_-modified compounds experienced a new decrease in compressive modulus; this property, however, for all the compositions remained well above the values observed for both filled (217 MPa, 353 MPa, and 363 MPa, respectively) and unmodified (104 MPa) un-aged specimens. Comparing these results with those found for filled-compounds cured with the other hardener (reported in Figure 4a), i.e., CaCO_3_-E5/TFF/100:26 systems, it can be noticed that the systems cold-cured with Z-1 keep values of compressive modulus generally lower than those observed for CaCO_3_-E5/TFF/100:26 compounds, irrespective to the time of immersion. Furthermore, the effect of a prolonged time in immersion (up to 10 months) is more pronounced for systems cured with Z-1.

The compressive maximum strength of CaCO_3_-filled E5/Z-1/100:12 systems appeared to be negatively influenced by the exposure to demineralised water (Figure 5b); on the other hand, the immersion time has a negligible effect on this property. Starting from 1 month of immersion, the maximum strength achieves and keeps values around 100 MPa, irrespective to the filler content. Once again, this value was not much lower than that measured on unaged E5/Z-1/100:12 control system, i.e., 113 MPa. The behaviour of compressive maximum strength found for CaCO_3_-filled E5/Z-1/100:12 systems aged in demineralised water resulted very similar with that observed for filled systems based on E5/TFF/100:26, reported in Figure 4b, even though for these latter systems the reductions in strength were found to be slightly influenced by the immersion times.

Finally, the strain at break measured during the compressive tests on CaCO_3_-modified E5/Z-1/100:12 systems immersed for different times in demineralised water was severely affected by this kind of aging procedure, as can be observed from the data reported in Figure 5c. This property was appreciably reduced after only 1 month of immersion and more than halved after 6 months, irrespective to the filler content. These reductions were in a certain extent recovered at the longest aging time, i.e., 10 months, although strain at break did not regain the initial (unaged) value for any of the CaCO_3_-contents analysed. The addition of filler, therefore, is not able to mitigate the adverse effects of water on compressive strain at break; a decrease of strain more than halved is observed for these systems if compared to the value measured on unaged unfilled E5/Z-1/100:12 (11%). In comparison with the systems cold-cured with the other hardener, i.e., CaCO_3_-filled E5/TFF/100:26, subjected to the same aging procedure (Figure 4c), the reductions in strain at break were found to be slightly lower by increasing the immersion times. However, after a 10-month aging period, very similar strain values were found for both groups of formulations, taking into account the spread of results.

### 3.3. Epoxy Samples Filled with Different Amounts of CaCO_3_ and Aged in Distilled Water 

**Figure 6:**
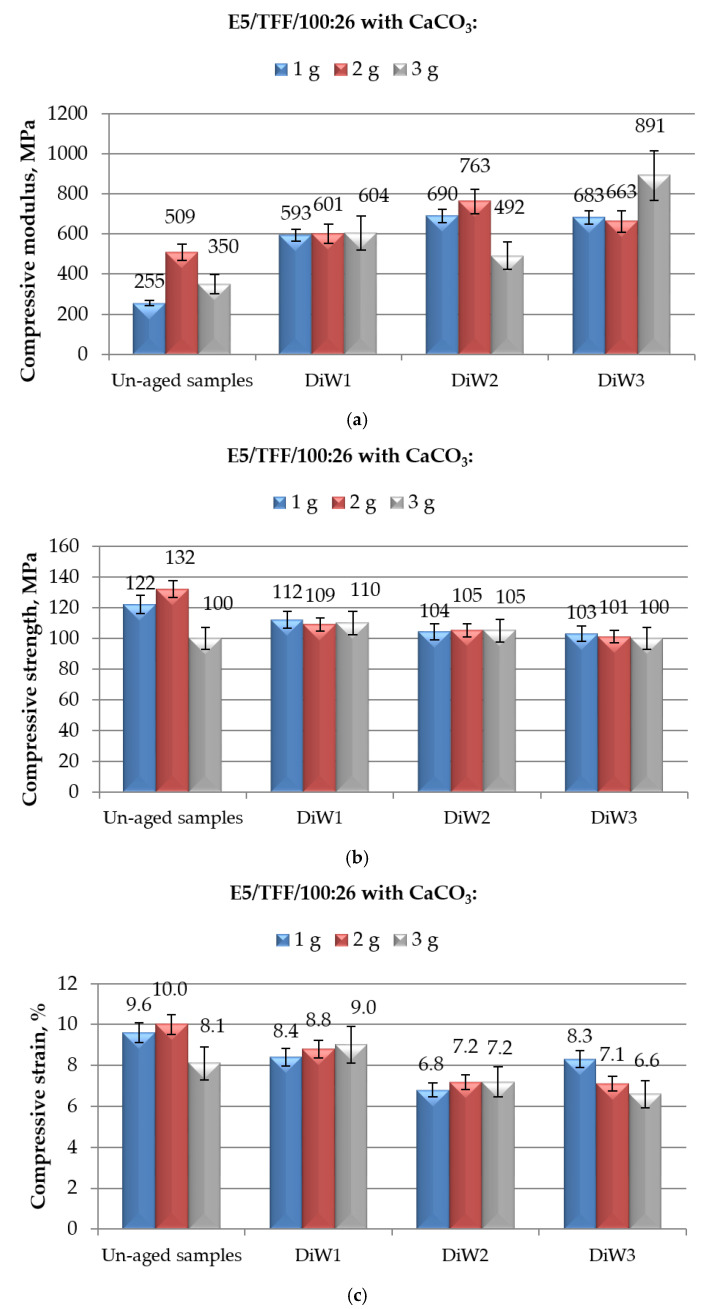
Results of mechanical (in compression mode) tests performed on E5/TFF/100:26 epoxy compounds filled with different amounts of CaCO_3_ and aged for different time spans (from 1 to 10 months) in distilled water: (**a**) compressive modulus; (**b**) compressive maximum strength; and (**c**) compressive strain at break.

**Figure 7:**
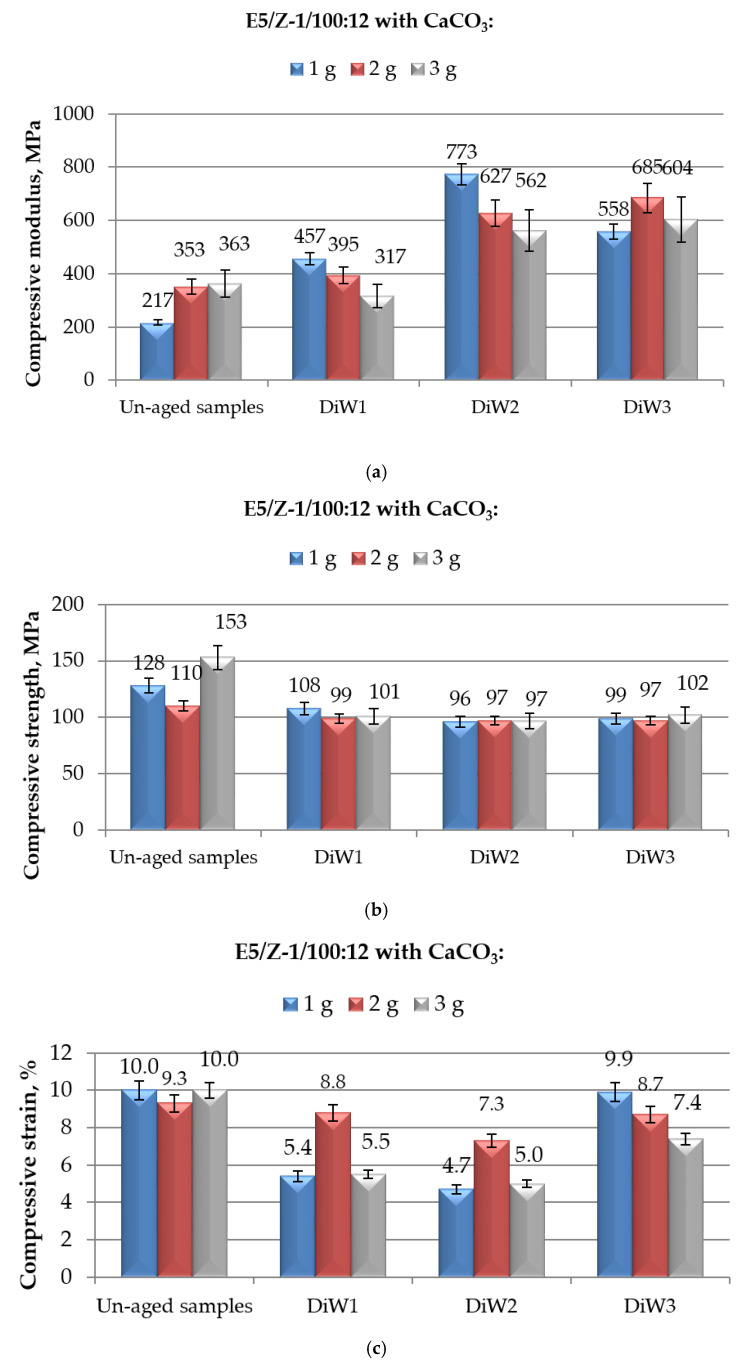
Results of mechanical (in compression mode) tests performed on E5/Z-1/100:12 epoxy compounds filled with different amounts of CaCO_3_ and aged for different time spans (from 1 to 10 months) in distilled water: (**a**) compressive modulus; (**b**) compressive maximum strength; and (**c**) compressive strain at break.

The effect of an immersion in distilled water on mechanical properties, measured in compression mode, of CaCO_3_-filled E5/TFF/100:26 and E5/Z-1/100:12 systems can be observed in Figure 6, and Figure 7, respectively.

Apart from an unavoidable dispersion of experimental results, the general behaviour of all filled systems aged in distilled water follows that of the same compounds aged in demineralised water (reported in Figure 4 and Figure 5, respectively), suggesting that the composition of water not containing mineral salts has an irrelevant effect on the mechanical (in compression mode) behaviour of cold-cured CaCO_3_-filled epoxy compounds.

Analysing in detail the data reported in Figure 6a, a substantial increase in compressive modulus was observed as consequence of the aging procedure in distilled water, irrespective to the filler content. Also in this case, the greatest increase (+155% over the same un-aged system) was observed for the system containing the highest amount (3 g) of calcium carbonate and aged for 10 months in distilled water. The growth in compressive modulus upon immersion in distilled water seems to proceed slightly slower with respect to what observed upon immersion in demineralised water (Figure 4a). However, all the obtained data are substantially greater with respect to the modulus found for unaged unfilled E5/TFF/100:26, i.e., 31 MPa, confirming the positive effect of the addition of calcium carbonate in epoxy adhesives exposed to aqueous environments.

The same slight decrease in the compressive maximum strength observed for CaCO3-filled E5/TFF/100:26 compounds as a consequence of the exposure to demineralised water (Figure 4b), can be observed when the same systems are aged in distilled water (Figure 6b), having the immersion time in distilled water a limited effect on the maximum strength only up to six months of aging. Referring to this aqueous environment, however, the effect of the filler content is almost negligible. Finally, also after a prolonged aging in distilled water, the CaCO_3_-filled E5/TFF/100:26 systems showed values of mechanical strength not much lower than the compressive strength measured for unaged E5/TFF/100:26 control (113 MPa).

In Figure 6c, the values of strain at break measured in compression tests on E5/TFF/100:26 systems loaded with different amounts of calcium carbonate are reported as a function of the immersion time in distilled water. By comparing these results with those observed for the same specimens immersed in demineralised water (Figure 4c), the reductions in strain at break are more gradual by increasing the aging time; moreover, the systems filled with lowest amounts (1 and 2 g) of CaCO_3_ experienced smaller reductions in strain after 10 months in distilled water. It is confirmed that the strain of aged CaCO_3_-filled E5/TFF/100:26 system is substantially reduced if compared to the value found for unaged E5/TFF/100:26 control system, i.e., 12%.

Passing to analyse the results of compression tests performed on CaCO_3_-filled E5/Z-1/100:12 systems aged up to 10 months in distilled water, reported in Figure 7, the general trends of modulus, maximum strength and strain at break are, again, comparable with those observed for the same systems aged in demineralised water (Figure 5), as well as for the CaCO_3_-modified E5/TFF/100:26 compounds exposed for the same periods of time in the same medium, i.e., distilled water (reported in Figure 6).

The compressive modulus (Figure 7a) experienced the greatest increase with respect to un-aged specimens at medium/high times of immersion in distilled water, i.e., of 256% for E5/Z-1/100:12/1CaCO_3_ exposed for 6 months, 94% and 66% for E5/Z-1/100:12/2CaCO_3_ and E5/Z-1/100:12/3CaCO_3_, respectively, both immersed up to 10 months. Comparing these results with those reported in Figure 5a for systems aged in demineralised water, it can be concluded that the reduction of compressive modulus at the longest immersion time (10 months) is much more limited, especially for systems containing 2 and 3 g of CaCO_3_. Once again, compressive modulus remained well above the values of this characteristic calculated for both filled (217 MPa, 353 MPa and 363 MPa, respectively) or unmodified (104 MPa) un-aged E5/Z-1/100:12 systems. Finally, comparing these results with those reported in Figure 6a for CaCO_3_-E5/TFF/100:26 systems, no specific trend can be deduced, since the effects of the kind of curing-agent depend on the calcium carbonate content and on the time of immersion in this aqueous medium.

The behaviour and numerical values of compressive maximum strength of CaCO_3_-filled E5/Z-1/100:12 systems aged in distilled water up to 10 months, observed in Figure 7b, are perfectly comparable with those of the same systems immersed in demineralised water (Figure 5b). The same considerations can be, then, repeated. The negative effects of distilled water on this property, with a negligible effect of the immersion time; a value of compressive maximum strength of aged systems filled with different amounts of calcium carbonate not much lower than that measured on unaged E5/Z-1/100:12 control system, i.e., 113 MPa. Again, the values of compressive maximum strength found for CaCO_3_-filled E5/Z-1/100:12 systems and those calculated for filled systems based on E5/TFF/100:26, reported in Figure 6b, all aged in distilled water, were very similar; the compressive strength values found for the systems cold-cured with Z-1 curing agent appears, again, less sensitive to the immersion times.

The compressive strain at break calculated for CaCO_3_-filled E5/Z-1/100:12 systems after different periods of immersion in distilled water are, finally, reported in Figure 7c. Also in this case, after a first severe reduction of strain upon 1 and 6 months of immersion, a new growth in this property is noted after 10 months, similarly to what can be seen for the same systems aged in demineralised water (Figure 5c). The only exception is represented by the system E5/Z-1/100:12/2CaCO_3_, whose value of compressive strain remained very close to that measured on un-aged system, almost irrespective to the immersion time. It is confirmed, therefore, that the addition of CaCO_3_ is able to reduce only to a limited extent the adverse effects of distilled water on compressive strain at break, that remained always much lower than the value measured on unaged unfilled E5/Z-1/100:12 (11%). The comparison of the strain at break values of CaCO_3_-filled E5/Z-1/100:12 systems with those found for the CaCO_3_-filled E5/TFF/100:26 ones, subjected to the same aging procedure (Figure 6c), confirms that the strain values of these latter systems are less affected by the permanence in this aqueous environment, especially for low to medium immersion times (1 and 6 months).

### 3.4. Epoxy Samples Filled with Different Amounts of CaCO_3_ and Aged in Spring Water 

**Figure 8:**
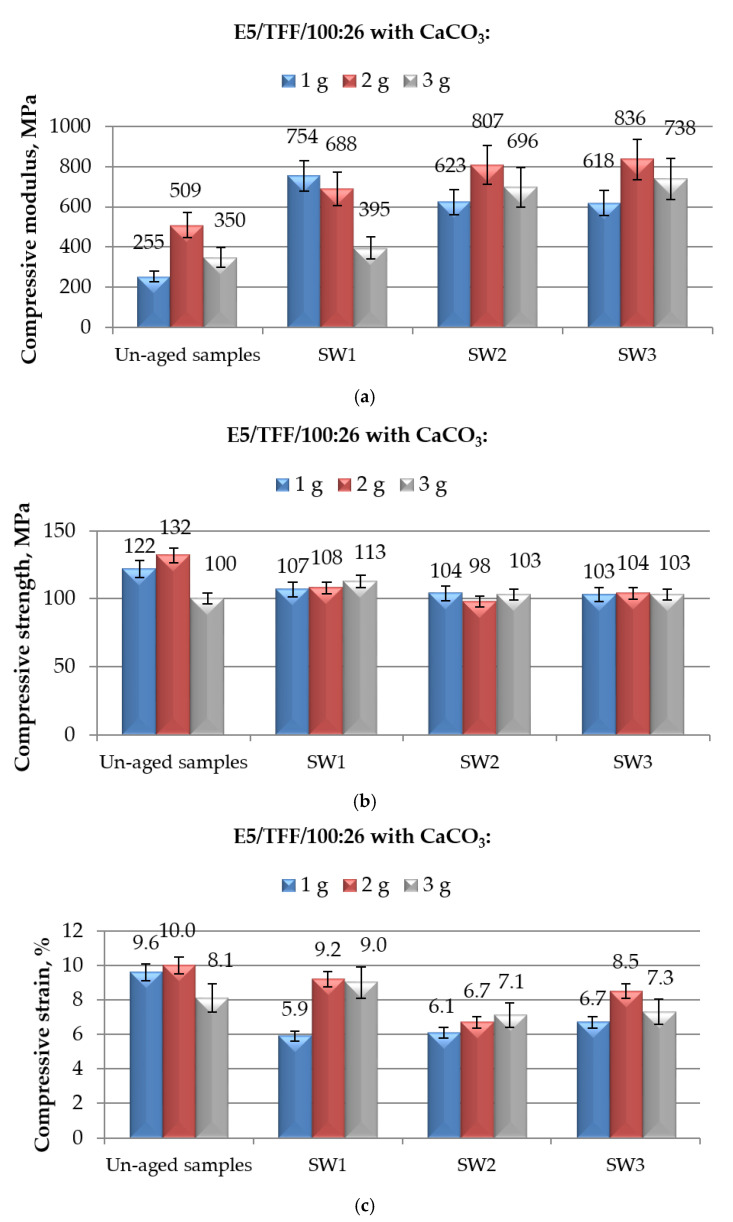
Results of mechanical (in compression mode) tests performed on E5/TFF/100:26 epoxy compounds filled with different amounts of CaCO_3_ and aged for different time spans (from 1 to 10 months) in spring water: (**a**) Compressive modulus; (**b**) compressive maximum strength; and (**c**) compressive strain at break.

**Figure 9:**
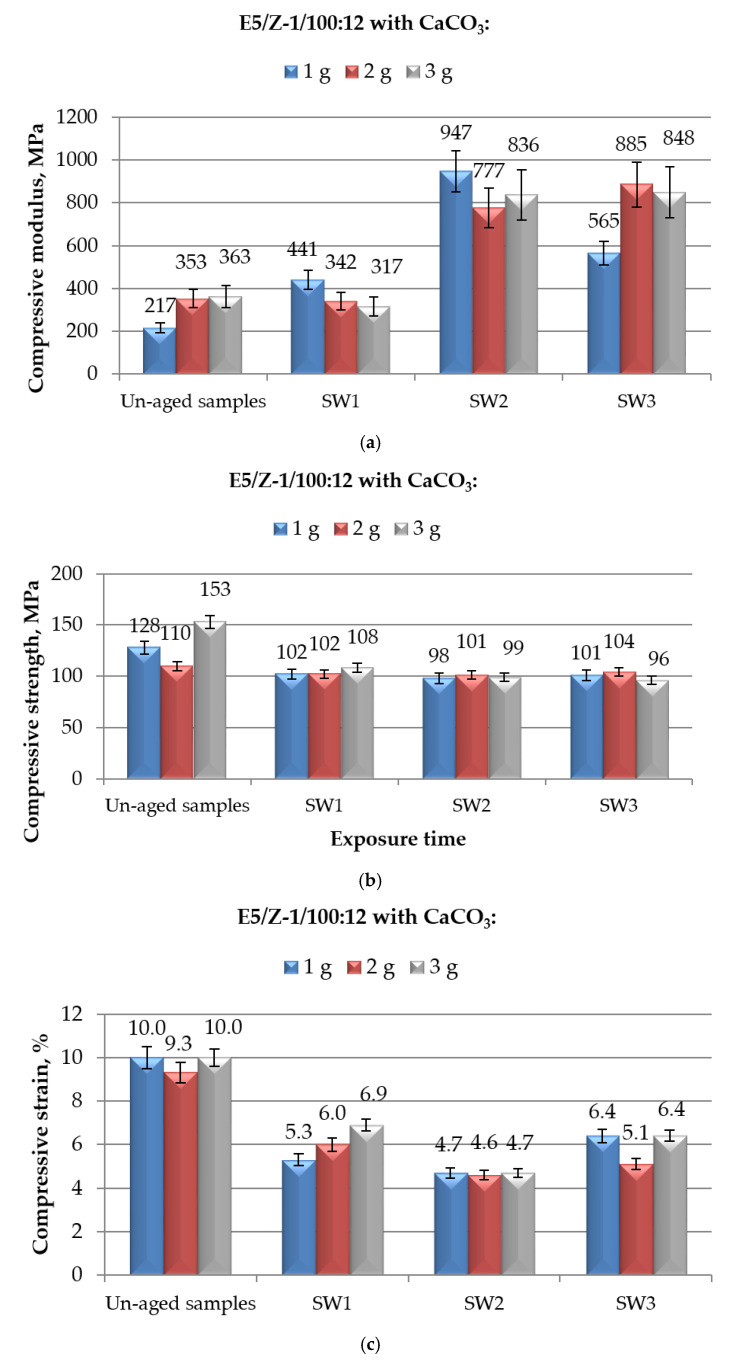
Results of mechanical (in compression mode) tests performed on E5/Z-1/100:12 epoxy compounds filled with different amounts of CaCO_3_ and aged for different time spans (from 1 to 10 months) in spring water: (**a**) Compressive modulus; (**b**) compressive maximum strength; and (**c**) compressive strain at break.

In the last part of this wide study, the effects of an immersion in spring water on mechanical properties, measured in compression mode, of CaCO_3_-filled E5/TFF/100:26 and E5/Z-1/100:12 systems were analysed; the results are presented in Figure 8, and Figure 9, respectively.

The general trends of compressive modulus, maximum strength and strain at break observed for CaCO_3_-filled systems aged in demineralised and distilled water are confirmed when the same compounds were immersed in spring water, with an increase in modulus, a slight decrease in strength and strong reductions in strain values as a consequence of the aging procedure.

A significant growth in compressive modulus of CaCO_3_-filled E5/TFF/100:26 system was recorded as consequence of an aging in spring water, as reported in Figure 8a. The growth was more regular by increasing the immersion time for the compounds containing 2 and 3 g of calcium carbonate, both achieving the highest value of compressive modulus after a 10-month aging procedure, with increases of 64%, and 111%, respectively, with respect to un-aged specimens; the behaviour of E5/TFF/100:26/1CaCO_3_ system resulted irregular, with an appreciable increase in modulus after 1 month of immersion and a subsequent slight reduction after 1 and 6 months. Comparing the numerical values of the compressive modulus reported in Figure 4a and Figure 6a for the same compounds exposed to demineralised water, and distilled water, respectively, it can be concluded that a prolonged aging (10 months) in spring water caused the lowest increases in this property. We highlight a high dispersion in the experimental results. Once again, the presence of filler was favourable, since the compressive modulus was well above the modulus found for unaged unfilled E5/TFF/100:26, irrespective to the filler content and aging time.

From the analysis of the compressive maximum strength data reported in Figure 8b, it can be repeated what already observed in the case of immersion in demineralised and distilled water (Figure 4b and Figure 6b, respectively), that is, the exposure to spring water brought about a slight decrease in the compressive maximum strength of CaCO_3_-filled E5/TFF/100:26 compounds; the exposure time had a limited effect on the maximum strength only up to 6 months of aging; the effect of the filler load is almost insignificant; irrespective to the filler content, the aged CaCO_3_-modified E5/TFF/100:26 compounds achieved values of mechanical strength not much lower than that recorded for unaged E5/TFF/100:26 control.

The values of compressive strain at break measured for filled systems aged in spring water, reported in Figure 8c, closely follow the same trend observed for the samples immersed in demineralised water, shown in Figure 4c, in terms of effect of filler and of exposure time. The system loaded with lowest (1 g) content of CaCO_3_ experienced an appreciable decrease in strain (39%) after 1 month of immersion, while it kept almost the same value at longer immersion times; after the shortest immersion time, the system E5/TFF/100:26/2CaCO_3_ display a value of compressive strain very close to that measured in its unaged state, while this value was appreciably reduced at longer immersion times, especially after six months; analysing, finally, the compound containing the highest amount (3 g) of filler, after an initial increase (+11%) in compressive strain upon a 1-month immersion in spring water, a reduction of strain around 10-12% with respect to unaged system was measured at longer aging times. Once again, the aging in spring water caused substantial reductions in compressive strain at break in E5/TFF/100:26 systems filled with small amounts of calcium carbonate.

The last analysed data refer to the results of compression tests performed on CaCO_3_-filled E5/Z-1/100:12 systems aged up to 10 months in spring water, reported in Figure 9. The behaviour observed for compressive modulus (Figure 9a), maximum strength (Figure 9b) and strain at break (Figure 9c) are comparable with that observed for the same systems aged in demineralised water (Figure 5) and in distilled water (Figure 7) as well as for the CaCO_3_-modified E5/TFF/100:26 compounds immersed in spring water (reported in Figure 8).

Referring to the compressive modulus (Figure 9a), it experienced an increase even greater that that observed upon exposure to demineralised (Figure 5a) and distilled (Figure 7a) water at the same immersion times, i.e., 6 months. A longer immersion time, i.e., 10 months, caused a reduction in this property, smaller for the filler contents equal to 2 and 3 g of CaCO_3_, remaining the compressive modulus also in this case well above the value calculated for unmodified un-aged E5/Z-1/100:12 control.

The values of compressive maximum strength measured on CaCO_3_-filled E5/Z-1/100:12 systems aged in spring water up to 10 months (Figure 9b) again follow what already observed for the same compound aged in the other aqueous environments (Figure 5b, and Figure 7b, respectively), with a certain reduction of this property after only 1 month of immersion and a minimal effects of filler content and aging times.

Finally, in Figure 9c the compressive strain at break data measured on CaCO_3_-filled E5/Z-1/100:12 systems after different periods of immersion in spring water are shown. This characteristic was confirmed to be severely reduced by the exposure of an aqueous environment, being the reductions in this property more evident at 6 months of immersion. Also in this case, a new increase in this property was recorded after 10 months of exposure, analogously to what observed for CaCO_3_-filled E5/Z-1/100:12 systems immersed in demineralised (Figure 5c) and distilled (Figure 7c) water for the same time. Very small appeared the effect of the filler content, irrespective to the aging time. From these latter results it is confirmed that upon addition of small amounts of CaCO_3_ it was not possible to appreciably limit the adverse effects of an aqueous environment on compressive strain at break of CaCO_3_-filled E5/Z-1/100:12 systems, since this value resulted much lower than that measured on unaged unfilled E5/Z-1/100:12.

## 4. Conclusions

In the present study, the effects of three aqueous environments on the mechanical properties, tested in compression mode, of an epoxy adhesive containing different amounts (from 1 to 3 g per 100 g of resin) of CaCO_3_ filler and cold-cured with two different curing agents, were evaluated. The capability of the selected filler to limit the detrimental effects of the aging exposures on mechanical properties of the epoxy resin was assessed at different immersion times, i.e., from 1 to 10 months. It was found that the effect of the curing agent was minimal on the compressive maximum strength and strain at break characteristics, measured on un-filled epoxy, while its choice affected the compressive modulus of pristine resin. As expected, the addition of calcium carbonate filler produced modifications in all the mechanical properties measured on un-aged epoxy systems, with increases in compressive modulus and maximum strength and reductions in strain at break, depending on the filler content. The aging regimes affected to a great extent the compressive properties of CaCO_3_-modified epoxy systems, the longer the immersion times, the greater the effects on the mechanical characteristics. The observed results can be justified by the plasticization phenomena occurring in the filled-epoxy during the aging in the aqueous media, not excluding an effect due to the continuation of the cold-cure, not completed after a curing process of a week at ambient temperature. These aspects, however, deserve further investigation. On the other hand, the kind of water employed in each aging regime, i.e., demineralised, distilled and spring water, had a minimal effect. Better performance against the aqueous environments were displayed by modified epoxy systems containing 2 and 3 g of CaCO_3_ per 100 g resin; such compounds, in fact, were able to keep compressive modulus and maximum strength greater than and close to, respectively, the same characteristics measured on unaged unfilled control epoxies. The results found in this study confirmed that it is possible to counteract the detrimental effects of an exposure to aqueous environments upon the addition of a cheap CaCO_3_ filler to cold-cured structural adhesives based on epoxy resins.

## Figures and Tables

**Table 1:** Epoxy resin properties.

Properties	Epoxy Resin–Epidian 5
Viscosity at 25 °C [mPa·s]	20,000–30,000
Density at 20 °C [g/cm^3^]	1.17
Curing time at 25 °C suggested by suppliers [days]	7–14
Physical state and colour	Viscous light-yellow liquid
Characteristic properties	Able to cure at room temperature (cold-curing epoxy); low shrinkage during curing; good mechanical properties; good chemical resistance (to lubricants, oils, diluted acids and lyes).

**Table 2:** Properties of the Mannich base (TFF) curing agent.

Properties	Curing Agent–Mannich Base
Viscosity at 25 °C [mPa·s]	max 10,000
Density at 20 °C [g/cm^3^]	1.15–1.20
Physical state and colour	Thick light-brown liquid
Characteristic properties	Fast reactivity with epoxy resin at ambient temperature (i.e., cold-cure); provides chemical resistance to the resin in most aggressive environment.

**Table 3:** Properties of the triethylenetetramine (Z-1) curing agent.

Properties	Curing Agent–Triethylenetetramine
Viscosity at 25 °C [mPa·s]	20–30
Density at 20 °C [g/cm^3^]	0.978–0.983
Physical state and colour	yellow/lightly green-yellow liquid
Characteristic properties	Able to cure epoxy at ambient temperature (i.e., cold-cure); easily soluble in water; can absorb water and CO_2_ from air; provides flexibility and impact strength to resins.

**Table 4:** Different, control and modified with calcium carbonate, epoxy compounds produced and analysed in the present study.

Formulation	Resin/Curing Agent [wt]	Amount of CaCO_3_ per 100 g Resin [g]
E5/TFF/100:26	100:26	0
E5/TFF/100:26/1CaCO_3_		1
E5/TFF/100:26/2CaCO_3_		2
E5/TFF/100:26/3CaCO_3_		3
E5/Z-1/100:12	100:12	0
E5/Z-1/100:12/1CaCO_3_		1
E5/Z-1/100:12/2CaCO_3_		2
E5/Z-1/100:12/3CaCO_3_		3

**Table 5:** Aging conditions.

No.	Aqueous Environment Type	Aging Time	Aging Code
1.	*Demineralised water*	1 month	DeW1
2.		6 months	DeW2
3.		10 months	DeW3
4.	*Distilled water*	1 month	DiW1
5.		6 months	DiW2
6.		10 months	DiW3
7.	*Spring water*	1 month	SW1
8.		6 months	SW2
9.		10 months	SW3

**Table 6:** Dissolved mineral component content in spring water (Gardinia Aleksandria Company, Domaslaw, Poland).

Components	Quantity [mg/L]
**Cations**	
Calcium Ca^2+^	52.10
Magnesium Mg^2+^	7.30
Sodium Na^+^	2.50
Potassium K^+^	0.86
**Anions**	
Hydrogen carbonate HCO_3_^−^	143.39
Sulfate SO_4_^2−^	27.78
Chloride Cl^−^	10.28
Fluoride F^−^	0.13
